# Midguts of *Culex pipiens* L. (Diptera: Culicidae) as a potential source of raw milk contamination with pathogens

**DOI:** 10.1038/s41598-022-16992-9

**Published:** 2022-08-01

**Authors:** Eslam Adly, Aml A. Hegazy, Mahmoud Kamal, Samah H. Abu-Hussien

**Affiliations:** 1 Entomology Department, Faculty of Science, Ain Shams University, Cairo, 11566 Egypt; 2 Food Science Department, Faculty of Agriculture, Ain Shams University, Cairo, 11241 Egypt; 3 Agricultural Microbiology Department, Faculty of Agriculture, Ain Shams University, Cairo, 11241 Egypt

**Keywords:** Applied microbiology, Pathogens, Biotechnology, Entomology

## Abstract

Despite their importance, only few studies focused on the mosquitoes borne microbial diseases, especially bacterial and fungal diseases, their vectorial capacity toward microorganisms, and their important role in raw milk contamination with pathogens in some unsanitary dairy farms. In Egypt, where *Culex pipiens* is the historical main vector of lymphatic filariasis, only few studies discussed the isolation of pathogens from the midguts of different stages of *C. pipiens*. This study aims to isolate and identify the pathogenic symbiotic microorganisms inside the midgut of adult female *C. pipiens* as well as investigate its ability to transmit their midgut pathogens to raw milk. A total of 750 field strain *C. pipiens *larvae of the second and third larval instars were collected from ponds water around the livestock farms in Mariotteya, Giza, Egypt, for microbial pathogen isolation and identification. All collected larval instars were transported to the laboratory at the Research and Training Center on Vectors of Diseases (RTC), Ain Shams University, where they were maintained for further studies. Six groups of *C. pipiens* were tested for the incidence of various pathogenic microorganisms in their midguts and their possibility to contaminate commercial sterilized milk. Traditional PCR assays and sequencing method detected and identified 16srRNA genes of the predominant hemolytic isolates from milk and midguts of female *C. pipiens*. The phylogenetic analyses of the obtained isolates were performed based on NCBI data. Three strains of *Bacillus anthracis* strain CPMESA 2021, *Staphylococcus warneri* strain CPSAME 2021, and *Bacillus cereus* strain CPSEMA 2021, which represent most food pathogens, were found in the midguts of *C. pipiens* and were submitted to the GenBank database with the accession numbers OK585071, OK576651, and OK585052, respectively. The isolation of these strains from mosquitoes raises contemporary issues concerning milk safety, such as bacterial isolates, the degree of the vectorial capacity of mosquitoes, milk production and processing conditions, and human pathogenicity. Such serious issues need further investigation.

## Introduction

Mosquitoes, the most dangerous dipterous flying insects, are considered the main reason for the loss of more than seven million human lives annually all over the world^1,2^. They have been categorized as one of the main important disease-transmitting vectors as they transmit various causative agents such as plasmodium, nematodes, and arboviruses, which are well known for causing many diseases like malaria, filaria, dengue, yellow fever, zika virus and chikungunya^3,4^. Among many genera and species of mosquitoes, the current study focuses on culicine mosquitoes. There are approximately 1000 culicine species worldwide responsible for transmitting different diseases such as Japanese encephalitis, lymphatic filariasis, West Nile fever, and Rift valley fever in human beings^5–8^. In Egypt, *Culex pipiens* is a widely distributed mosquito species, which is considered the historical main vector of lymphatic filariasis^4,9,10^. Also, it has been suspected and incriminated in the transmission of the Rift Valley fever and the bancroftian filariasis in the Nile Delta of Egypt^3,4^.

Symbiotic bacteria are naturally found in the midgut of many insects providing them with necessary nutrients and helping them in food digestion. On the other hand, they affect the insect’s metamorphosis, reproduction, and their role in insect speciation^11,12^. For mosquitos, these symbiotic bacteria do not only affect the insect itself but also help in activating and improving the innate immune system of the mosquito insect to resist the infection against its parasites^13^. Furthermore, these microbiotas inside the mosquito’s midgut may alter the development and transmission of pathogens in which the adult female mosquito could transmit pathogens to humans during bloodsucking. It is necessary to characterize the bacterial fauna of adult female *C. pipiens* due to its importance as a vector of *Wuchereria bancrofti* which causes Lymphatic filariasis in Egypt^4^. Many researchers have studied the microflora in the midgut of *C. pipiens* at all developmental stages but didn’t identify them by molecular assays. Moreover, they didn’t study the ability of mosquitoes to transmit pathogens to the surrounding environments, especially in dairy processing^14–18^. Microbial contamination of milk in the value chain can originate from a diseased cow, unhygienic milking practices, poor personal hygiene, unsanitary utensils and/or milking equipment, and water supplied in sanitary activities^19^.

Studies on insect-borne bacterial diseases are often neglected, except for some bacterial diseases transmitted by ticks^20^. Information about the possibility of mosquitoes to transmit bacterial disease agents as well as the vectorial capacity of mosquitoes is very poor. Only few studies dealt with the biodiversity of symbiotic bacteria in mosquitoes, like *Wolbachia* spp., and the mechanical transmission of tularemia (*Francisella tularensis*) to humans by mosquitoes^21,22^. Other studies focus on the old known bacteria *Bacillus anthracis*, the causative agent of anthracis, which has been isolated from *Aedes aegypti* and transmitted mechanically by *A. aegypti*^23^. However, the most important research was done in 2015 by a group of scientists who discovered that *Anopheles gambiae* is the potential vector of *Rickettsia felis*, the main causative agent of flea-borne spotted fever^16^. Also, they isolated *R. felis* from cotton that was used in feeding mosquitos with sucrose meal in the laboratory. These results improve the possibility of mosquitoes in transmitting bacterial diseases not only by mechanical methods but biologically as well^24^. Furthermore, their discoveries raised an important question: can the mosquitoes transmit bacterial pathogens mechanically or biologically to milk as an essential food source for humans when they accidentally fed on it in farms of livestock or in houses?

This research aims to isolate and identify the pathogenic symbiotic bacteria inside the midgut of adult female *C. pipiens* mosquitoes in Egypt. Moreover, this study investigates the possibility of these bacteria being transmitted from their midguts to milk during feeding which can affect the safety and quality of raw milk.

## Materials and methods

### Collection and maintenance of mosquito larvae

A total number of around 750 larvae of the field strain of *C. pipiens* were collected in the second and third larval instars from stagnant water bogs around the livestock farms in Mariotteya, Giza, Egypt. All collected larval instars were transported to the laboratory at RTC, Ain Shams University, for maintenance and studies. All collected larvae were washed three times in sterilized distilled water to remove any possible contamination.

Rearing of larvae was done according to the standard techniques as described earlier by Ukubuiwe et al.^25,26^ with minor modifications. Three plastic-enamel trays were filled with two liters of distilled water each. Larvae were placed at the ratio of 250 larvae/tray. Larvae feeding was carried out by sprinkling fish feed (Tetra-/Min, Germany) on the trays’ water surface at the rate of 0.80 mg/250 larvae every other day. Water in trays was changed on the alternative days to prevent scum formation and to ensure hardness level till emerging. Once pupation has occurred, all released pupae were collected daily and placed in another plastic-enamel tray (5 cm height and 20 cm diameter) half-filled with distilled water. All trays were kept in adult-holding cages until the adult insects’ emergency.

### Feeding female adult mosquitoes

For feeding the newly emerged female adult mosquitoes, sucrose solution (10%), commercial sterilized milk, and a pigeon were prepared in triplicates. All newly emerged female adult mosquitoes were divided into three groups and subjected to three feeding strategies. Group A (10% Sucrose-fed females) included the newly emerged adults which were fed on 10% sucrose solution to maintain their activities. Group B (Sterilized milk-fed females) included the newly emerged adults which fed on sterilized commercial milk to investigate their potentiality for bacterial transmission. Group C (Blood-fed females) included the *C. pipiens* adults which fed on the blood of pigeon birds to obtain nutrients needed for mating and giving birth to *C. pipiens’* new generations^27^.

### Processing of mosquito samples

*Culex pipiens* mosquitoes were sorted into three groups based on stages and age post-emergence (4th larvae instar, newly emerged adult females, and newly emerged adult males) and adult females were divided into three groups based on different nutritional treatments (10% Sucrose-fed females, commercial sterilized milk-fed females, and Blood-fed females).

Each group included 30 individuals from the selected stage. Processing of mosquitoes’ samples for isolation of pathogenic symbiotic bacteria was done by immobilizing and killing all the individuals by cold shock by exposing them to − 20 °C for 2 min to avoid hemolymph excretion.

Concerning adult processing: wings and legs were gently removed, and samples were individually surface-sterilized for 30 s in a micro centrifugal tube containing 250 µl 70% ethanol to remove their outer waxy layer. Then, they were rinsed twice with cold freshly prepared 250 µl phosphate-buffered saline (PBS) pH 7.4 on a cold microscopic slide. Under a research stereomicroscope (Carl Zeiss; serial No. 2004000736), each *C. pipiens* was cut at the abdomen’s end, and the midgut was gently teased out with a mounted needle, while a gentle pressure was applied to the abdomen. The midgut samples were trimmed of adherent tissues and then transferred immediately to small Eppendorf® tubes (5 ml) containing 500 µl of ice-cold sterile PBS. (pH 7.4) and homogenized with a sterilized micro mortar. Tenfold serial dilutions from 10^−2^ to 10^−7^ were prepared for diluting the homogenized lysate using PBS tubes (900 µl).

For larvae processing: heads and siphons were removed gently, and the rest of the body was collected and sterilized in the same way as adult samples.

The final discard was cultivated for bacterial screening to ensure no contamination. All the six groups’ samples: (larvae, newly emerged adult females, newly emerged adult males, 10% Sucrose-fed females, Sterilized milk-fed females, and Blood-fed females) were screened for the presence of the midgut bacteria using the plate count technique.

### Collection and sampling for mosquito living habitats and raw milk samples

All maintained larvae were transported to the microbial inoculant center (MIC) and Cairo Microbiological Resources Center (Cairo MIRCEN), Fac., Agric, Ain Shams University. Three feeding and living habitats were selected for detecting the transmission of the midgut’s microbiota: Pond water’ habitat, and commercial sterilized milk before and after mosquito’s feeding. For pond water sampling, three collected water samples were obtained from the same larval collecting sites at stagnant water bogs around the livestock farms in Mariotteya, Giza, Egypt as described above in the mosquito collection and maintenance section. For milk sampling, 5 samples of commercial sterilized milk were collected from local markets as control treatment against commercial sterilized milk used as a feeding medium for female Adult *C. pipiens*. All samples were packed individually in sterilized glass bottles, transported to the lab, and stored at 4 °C for further studies.

### Isolation of midgut bacteria of *C. pipiens*

As mentioned earlier, the mosquitoes were divided into six groups: larvae, newly emerged adult females, newly emerged adult males, 10% Sucrose-fed females, sterilized milk-fed females, and Blood-fed females. A total of 180 mosquitos’ specimens (30 individuals × 6 groups) of each group were collected, processed and surface sterilized using ethyl alcohol 70% again for 5 min followed by washing twice with sterilized water to get rid of any possible contamination. Samples were taken from each group and minced under aseptic conditions. Tenfold serial dilutions were prepared in 0.1% peptone. A 100-μl volume from each dilution was plated on a sterile medium for each microbial pathogen individually as described in the bacterial isolation section. These media were incubated at 37 °C for 24–48 h. Microbial growth was assessed based on the total number of colony-forming units (CFUs). Bacterial colonies were distinguished morphologically (i.e., shape, size, color, margin, opacity, and elevation). Morphologically distinct colonies were selected from primary plates for repeated subculture on nutrient agar plates until a pure colony was obtained.

#### Total bacterial count

Sample dilutions were plated in triplicates on tryptone glucose yeast extract agar (TGYA)^28^. Plates were incubated at 25 °C for 48–72 h. and colony counts per 50 mosquitoes were assayed.

#### Blood hemolytic bacteria

Samples of mosquitoes’ groups and mosquito feeding and living habitats were inoculated on Blood agar^28^. Incubation was done at both aerobic and under anaerobic conditions using CO_2_ incubator at 37 °C for 24 h. The plates were examined for the blood hemolytic colonies and colony counts per 50 mosquitoes were assayed. Colonies surrounded by green zone were Alpha hemolytic colonies, beta-hemolytic colonies were detected by clear zones around colonies, and gamma hemolytic colonies are non-blood hemolytic.

#### Coliform

Violet red bile agar with added lactose (VRBL) medium^28^ was used as a selective medium for the detection and enumeration of coliform bacteria. Plates were incubated at 37 °C for 48 h. Colonies with purple color were counted.

#### Salmonella

Xylose Lysine Deoxycholate agar medium (XLD agar)^28^ was used for the isolation of salmonella. First, pre-enrichment was made in selenite broth for 24 h. at 37 °C, then 1 ml of 24 old culture was inoculated on XLD for 24 h, and black colonies were counted.

#### Shigella and Escherichia coli

Salmonella-shigella agar (SS agar)^28^ was used in the isolation of *Shigella* sp. and *E. coli.* Incubation was done at 37 °C for 48 h. Black and yellow colonies were counted for *Shigella* and *E. coli*, respectively. Positive *E. coli* isolates were confirmed by subculturing on EMB solid medium at 37 °C for 24 h. Black colonies with greenish metallic pigment were picked up and sub-cultured on MacConkey liquid medium at 44 °C for 24 h. positive acid and gas producers were allowed to grow on Indole medium^28^ and incubated at 37 °C for 24 h. Positive isolates with red ring formation on the medium surface were selected for the hemolytic test.

#### Staphylococcus aureus

Paired parker agar medium^28^ was used for isolation of staphylococci. At pouring, 0.01% (w/v) of potassium tellurite (K_2_O_3_) was added, and then plates were incubated for 24–48 h at 37 °C. Black rough colonies with yellow halo were counted.

#### Total fungi and yeasts

Oxytetracycline medium^28^ was prepared. Plates were incubated for 5–7 days at 25–28 °C. Fungal and yeast colonies were counted.

#### Bacillus cereus

Mannitol yolk polymyxin B agar (MYPA) medium^28^ was prepared. Plates were incubated for 24–48 h at 30 °C pink colonies were positive and counted using plate count assay.

### Isolation and identification of pathogenic bacteria from milk samples

All milk samples were analyzed for previous pathogenic bacteria transmitted as a result of feeding females *C. Pipiens* according to ISO standards (enumeration of the total bacterial count, enumeration of coliform, detection of *Salmonella*, enumeration of *E. coli*, enumeration of *Staph aureus*, enumeration of yeasts and molds and enumeration of *Bacillus cereus* according to ISO 4833-1:2013, ISO 4832: 2006, ISO 6579-1: 2017, ISO 16649-2:2001, ISO 6888-1:1999, ISO 21527-1:2008 and ISO 7932:2004 respectively). The isolates were identified according to DNA and 16s rRNA as follows.

### Isolation of DNA and 16s rRNA gene amplification of the most dominant pathogens affecting milk safety

For isolation of DNA, QIA amp DNA mini kit (QIAGEN GmbH, Hilden, Germany) was used for each pure culture as described by the manufacturer’s instructions. Universal primers of 27F (5′ AGAGTTTGATCCTGGCTCAG 3′) and 1492R (5′ TACG GCTACCTTGTTACGACTT 3′) was used to amplify DNA which targets the 16S rRNA gene sequences. To perform the Polymerase Chain Reaction (PCR), a reaction mixture with total volume of 25 μl was prepared containing 1 × PCR buffer solution (Invitrogen), 0.5 μm of each primer, 2.5 mM MgCl_2_, 200 ng of purified DNA, 0.2 mM dNTPs, and 0.3 units of Taq polymerase (Invitrogen) against negative control treatments containing PCA media and ddH_2_O. Cycles of amplification for all samples started with the initial denaturation cycle at 94 °C for 10 min, followed by 35 denaturation cycles at 94 °C for 30 s, annealing cycles at 55 °C for 30 s, and extension cycles at 72 °C for 1 min. The final extension cycle was at 72 °C for 8 min. Agarose gel (1%) containing ethidium bromide using a UV transilluminator was prepared for the visualization of the amplified product. Purification of PCR products was done using the QIA quick PCR Purification Kit (Qiagen). The purified products were sent to Macrogen, South Korea (Macrogen Inc., 1001, 254 Beotkkot-ro, Geumcheon-gu, Seoul, Republic of Korea) for 16S ribosomal RNA partial gene sequencing by the Sanger method. The resultant sequences were compared to the databases of the GenBank (www.ncbi.nlm.nih.gov/BLAST) for confident sequence analysis, seq-match and sequences similarity using similarity check tools.

According to GenBank identification of isolates, sequence comparison of their classification at genus and species level was done; 98% or higher sequence identities with the GenBank data entries were suggested for species delineation^29^. All confirmed sequences were submitted to GenBank.

### Sequence analysis and distance tree construction

The nucleotide FASTA sequence was submitted to NCBI GenBank under accession numbers (OK585071, OK576651, and OK585052) and NCBI database BLAST. The distance tree for the sequences was viewed using NCBI TREEVIEWER and constructed neighbor-joining phylogenetic cladogram tree with the identification based on sequence similarities^30^.

## Results

### Bacterial diversity in pond water: the habitat of collected mosquito larvae

**Figure 1:**
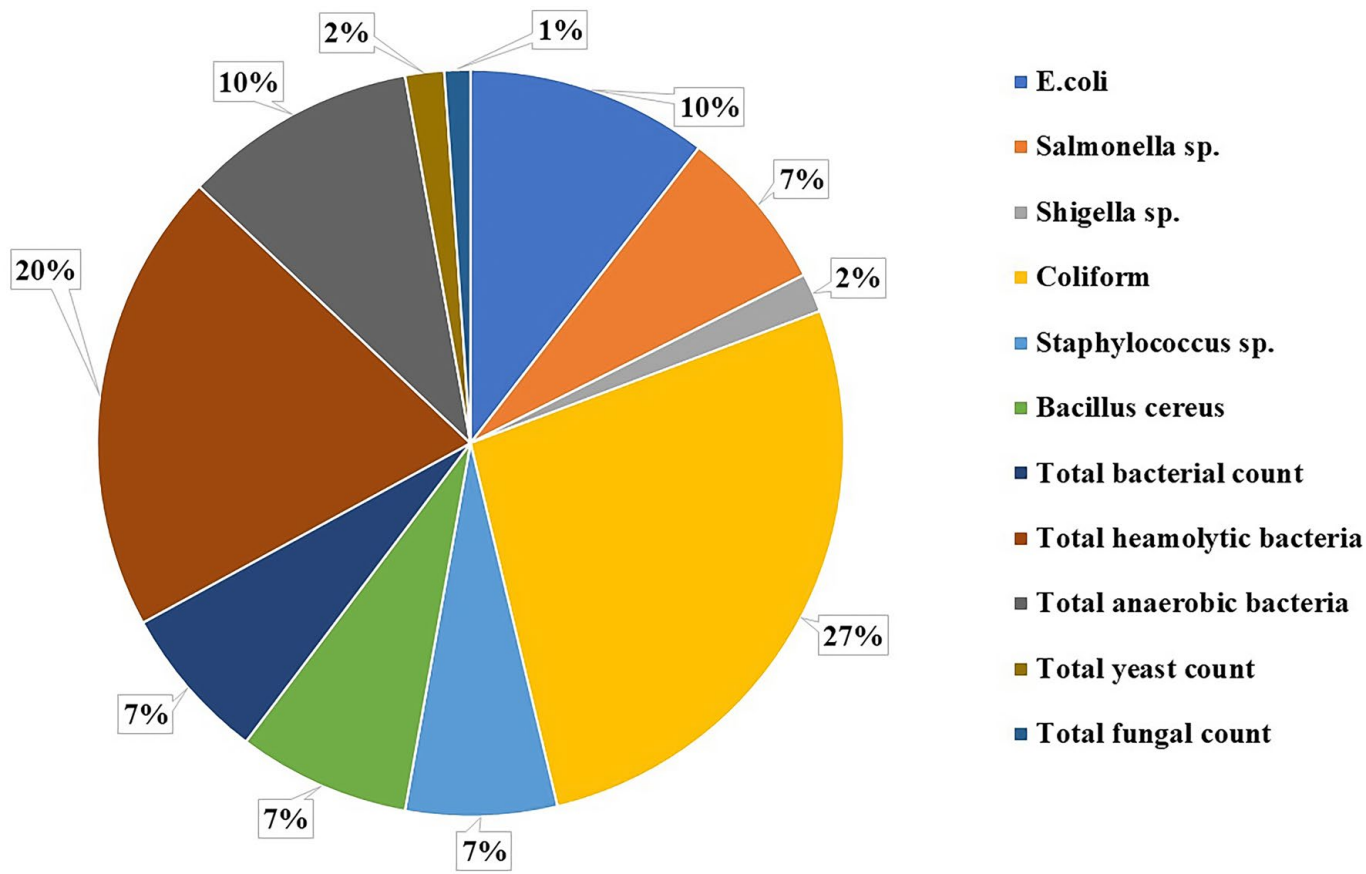
Incidence of microbial groups and strains in Pond water’ habitat of collected mosquito’ larvae around the livestock farms in Mariotteya, Giza, Egypt.

In this case study, a total of 15 water samples from stagnant water bogs around the livestock farms in Mariotteya, Giza, Egypt, were collected and the collection sites were analyzed as shown (Fig. 1). 65 bacterial and fungal isolates were detected from all pond water samples. Bacterial culturing results on specific media revealed that *E. coli*, *Shigella*, *Salmonella*, *Staphylococcus*, *Bacillus cereus* found in large incidence percentage reached 23%, while total fungi and yeasts represented only 3% of the total bacterial and fungal flora. Total anaerobes and total hemolytic bacteria represented 10% and 20% of the total microflora, respectively. Data clearly showed that coliform had the greatest incidence with more than 27% in all pond water samples.

### Microbial isolates from midguts of mosquitoes

A total of 50 bacterial isolates were obtained during this study from different mosquito instars fed on different media. All obtained isolates were stored at 4 °C and sub-cultured at monthly intervals for the hemolysis test. The blood hemolytic bacteria were then identified on the basis of molecular analysis methods. All *E. coli* isolates were maintained at 4 °C and sub-cultured at monthly intervals for the total coliform fecal test.

#### Microbial diversity in different stages of *C. pipiens*

**Figure 2:**
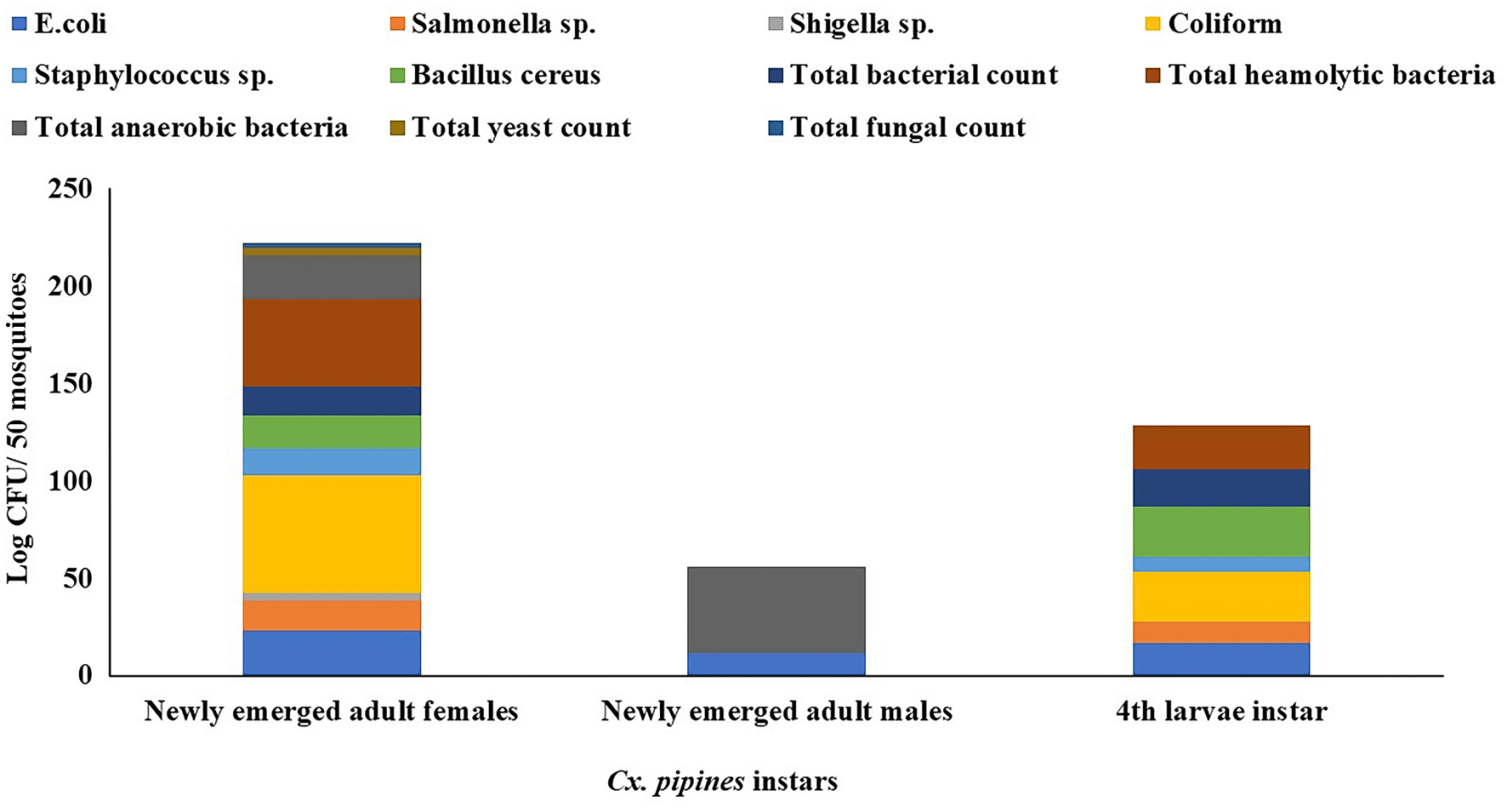
Relative abundance of bacterial genera in the midgut of *C. pipiens* collected from ponds water around the livestock farms in Mariotteya, Giza, Egypt and reared in the laboratory.

All newly emerged adult females, males, and the 4th larval instar were sampled as described above and tested for the presence of different microbial groups. Data in Fig. 2 shows that the newly emerged adult males have low microbial counts of only *E. coli* and total anaerobes reached 15 and 52 CFU/50 mosquitoes. While, the newly emerged adult females had the most predominant isolates of *E. coli*, *Salmonella* sp., *Staphylococcus* sp., *Bacillus cereus,* and the hemolytic bacteria and low incidence of *Shigella*, total yeasts, and fungi.

**Figure 3:**
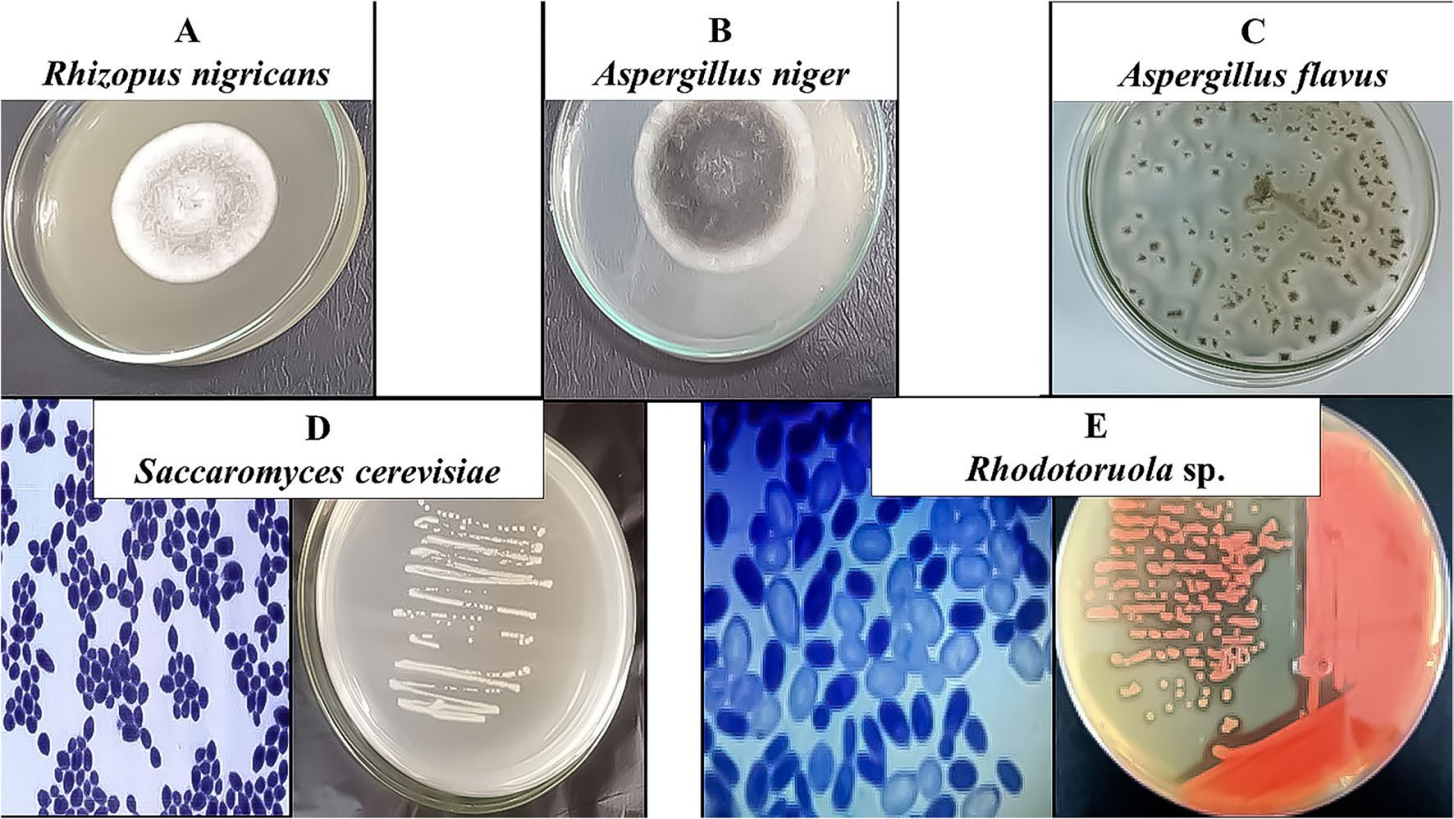
Morphological characters of some fungal isolates obtained from the midgut of *C. pipiens* collected from ponds water around the livestock farms in Mariotteya, Giza, Egypt and reared in the laboratory.

Data in Table 1 and Fig. 3 revealed that the *C. Pipiens* mosquitoes carry and harbor pathogenic and spoilage fungi during their developmental stages. Six isolates were obtained and were examined microscopically. The most predominant fungal strains were *Rhizopus nigricans*, *Aspergillus niger*, and *Aspergillus flavus* in the adult’s female, males, and larval stages. Moreover, yeast isolates were identified morphologically as *Saccharomyces cerevisiae* and *Rhodotorula* sp. All fungal and yeast isolates were found in all developmental stages of *C. pipiens*.

**Table 1:** Fungal diversity in the midgut of *C. pipiens* collected from the field in Mariotteya, Giza, Egypt, and reared in the laboratory.

Isolate no	Suggested genus	Larvae	Female	Male
1	*Rhizopus nigricans*	+Ve	+Ve	+Ve
2	*Aspergillus niger*	+Ve	+Ve	+Ve
3	*Aspergillus flavus*	+Ve	+Ve	+Ve
4	*Saccharomyces cerevisiae*	+Ve	+Ve	+Ve
5	*Rhodotorula* sp*.*	+Ve	+Ve	+Ve

#### Microbial diversity in midgut of nourished female Adult *C. pipiens*

**Figure 4:**
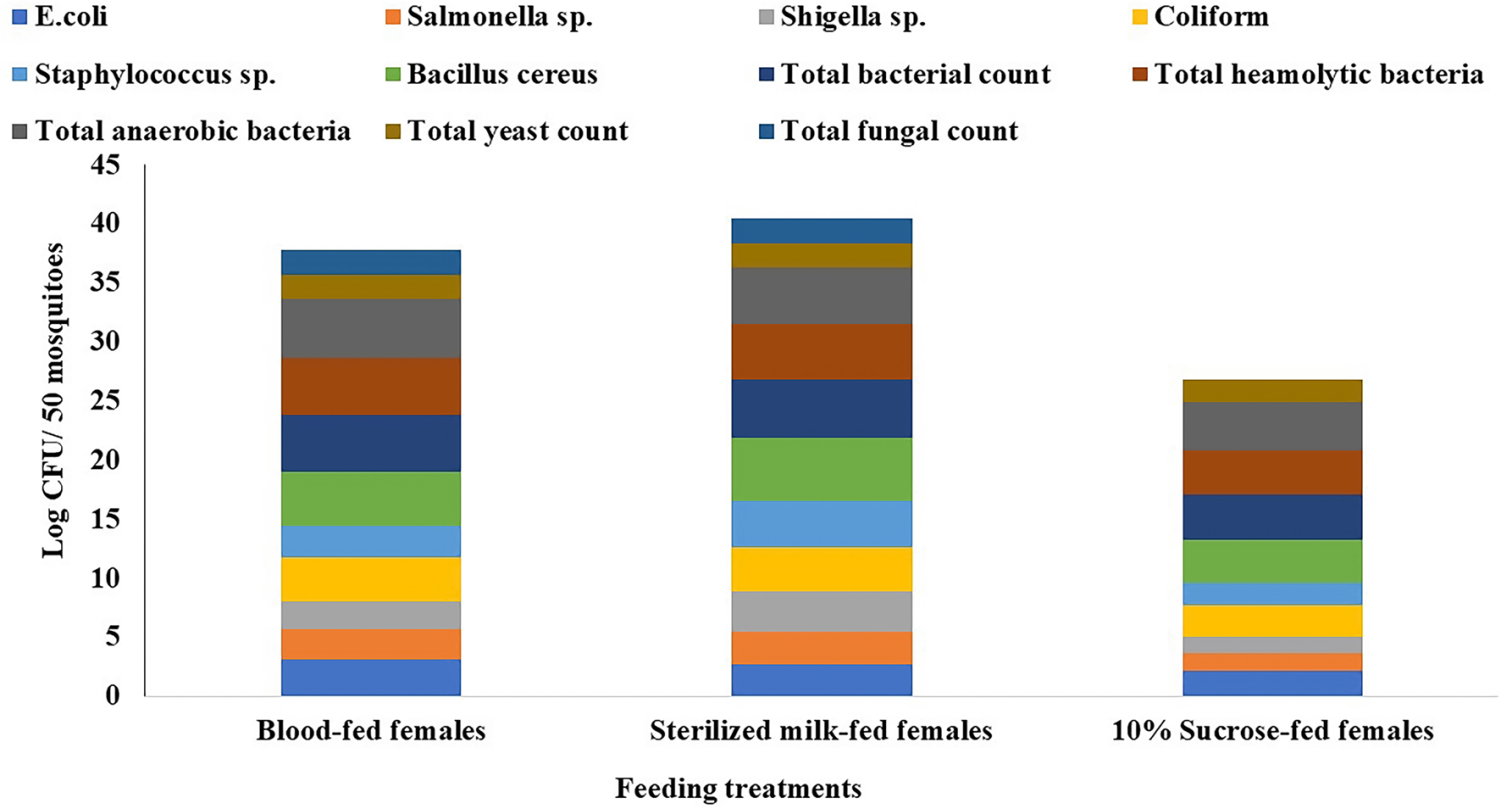
Microbial count (Log CFU/50) of different microbial genera in the midguts of adult females of *C. pipiens* fed on different feeding sources after 24 h. of incubation at suitable temp.

To study the microbial flora in the midguts of *C. pipiens* females during their feeding, three feeding materials were selected as blood, sterilized milk, and 10% sucrose solution. Data in (Fig. 4) shows that when adult females were fed on sterilized milk, they had the most variations of total microbial groups inside their guts other than the blood-fed females. While the sucrose-fed females had the lowest microbial incidence in their midguts. The most predominant bacteria in all of the blood-fed, sterilized fed and 10% sucrose solution-fed females were *Staphylococcus* sp., *Bacillus cereus*, total hemolytic bacteria, and the total anaerobes. coliform group, *Salmonella*, *Shigella,* and *E. coli* had a low presence when compared to the newly emerged adult females.

#### Bacterial isolates from milk used as a feeding source for female *C. pipiens*

**Figure 5:**
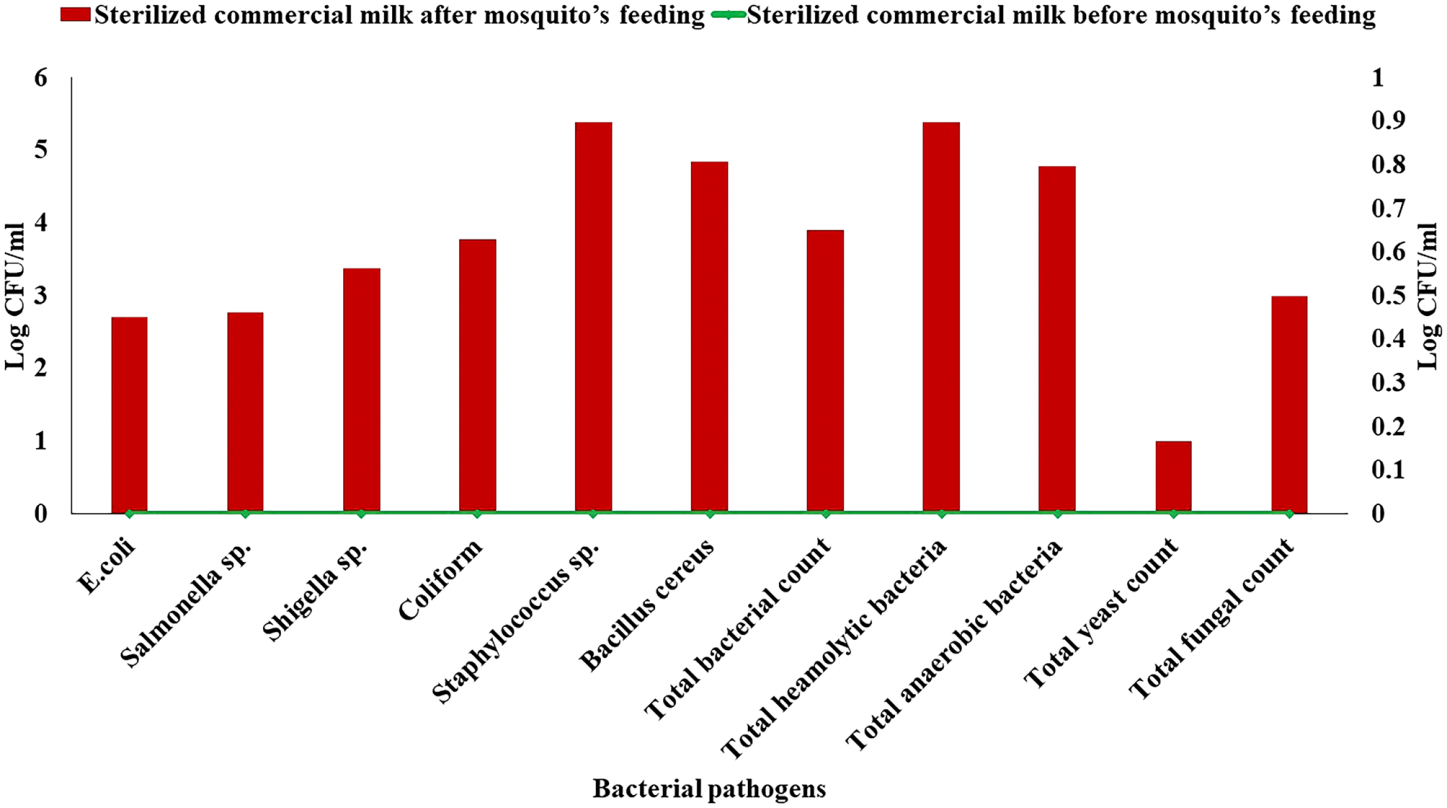
Microbial count (Log CFU/ml) of different microbial genera in the feeding milk habitat of *C. pipiens* after 24 h. of incubation ﻿at suitable temp.

To confirm that *C. pipiens* have the ability to transmit their midgut microbes into the feeding medium as sterilized milk. Newly emerged adult females in the laboratory were allowed to feed on sterilized milk for 24 h under aseptic conditions. Data in Fig. 5 proves that all microbial pathogens, especially *Staphylococcus* sp., *Bacillus cereus*, total hemolytic bacteria, and total anaerobes were transmitted into the sterilized milk samples. This indicates a new source of milk contamination that threatens the safety and quality of milk samples. Fifty isolates were picked up and sub-cultured, then maintained at 4 °C for detecting their ability to produce hemolysins which play an important role as a virulent factor in most pathogens.

**Figure 6:**
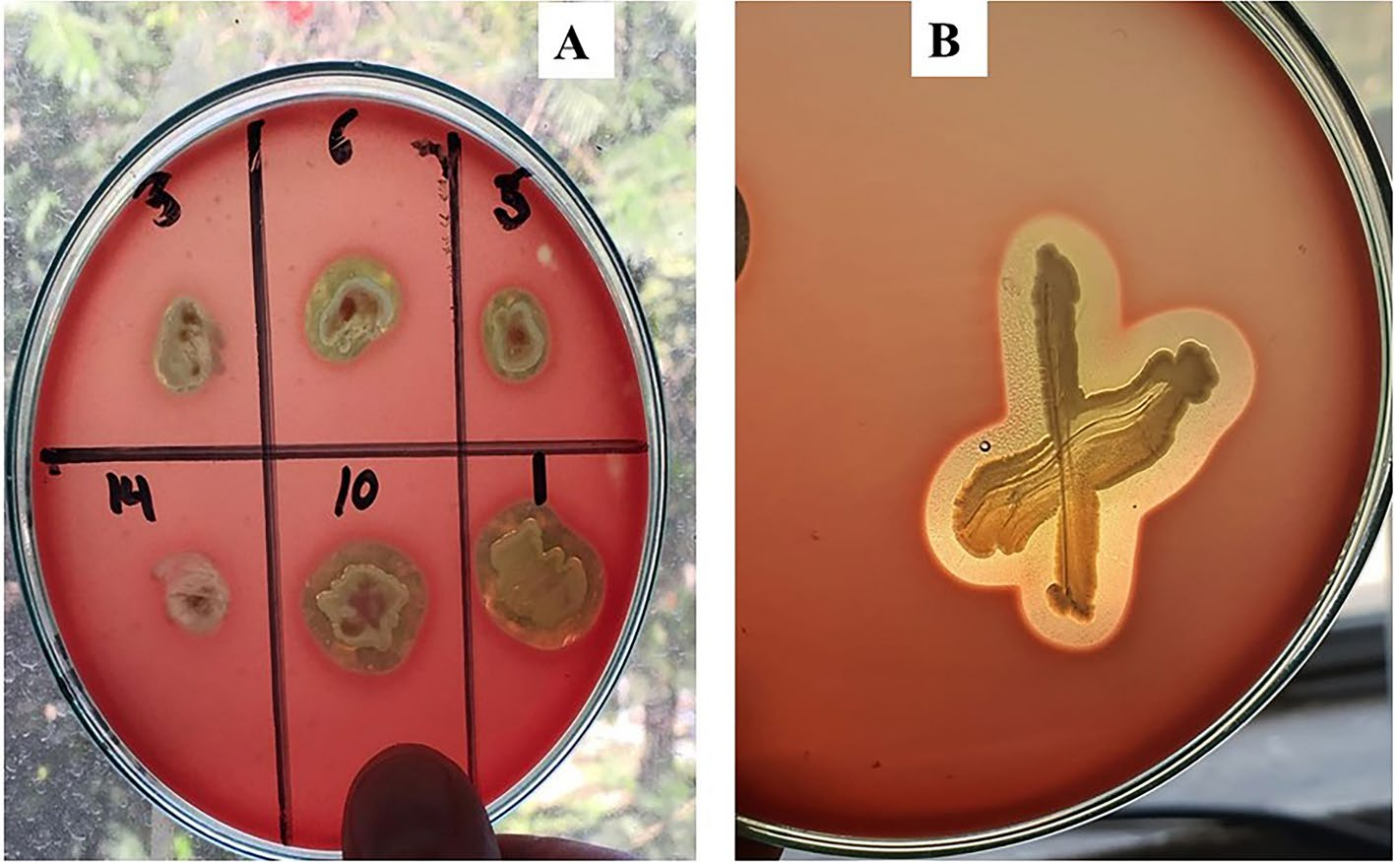
Blood hemolysis test of the isolated bacteria in the feeding milk habitat of *C. pipiens* after 24 h. of incubation ﻿at suitable temp.

Out of the fifty isolates, 20 isolates were alpha-hemolytic bacteria, 15 isolates were beta-hemolytic and 5 of them were gamma hemolytic bacteria as shown in Fig. 6. Among the hemolytic bacteria, *Staphylococcus* sp., *Bacillus cereus,* and anaerobic hemolytic *bacilli* were the dominant beta-hemolytic bacteria. Also, *E. coli* was a positive producer.

**Figure 7:**
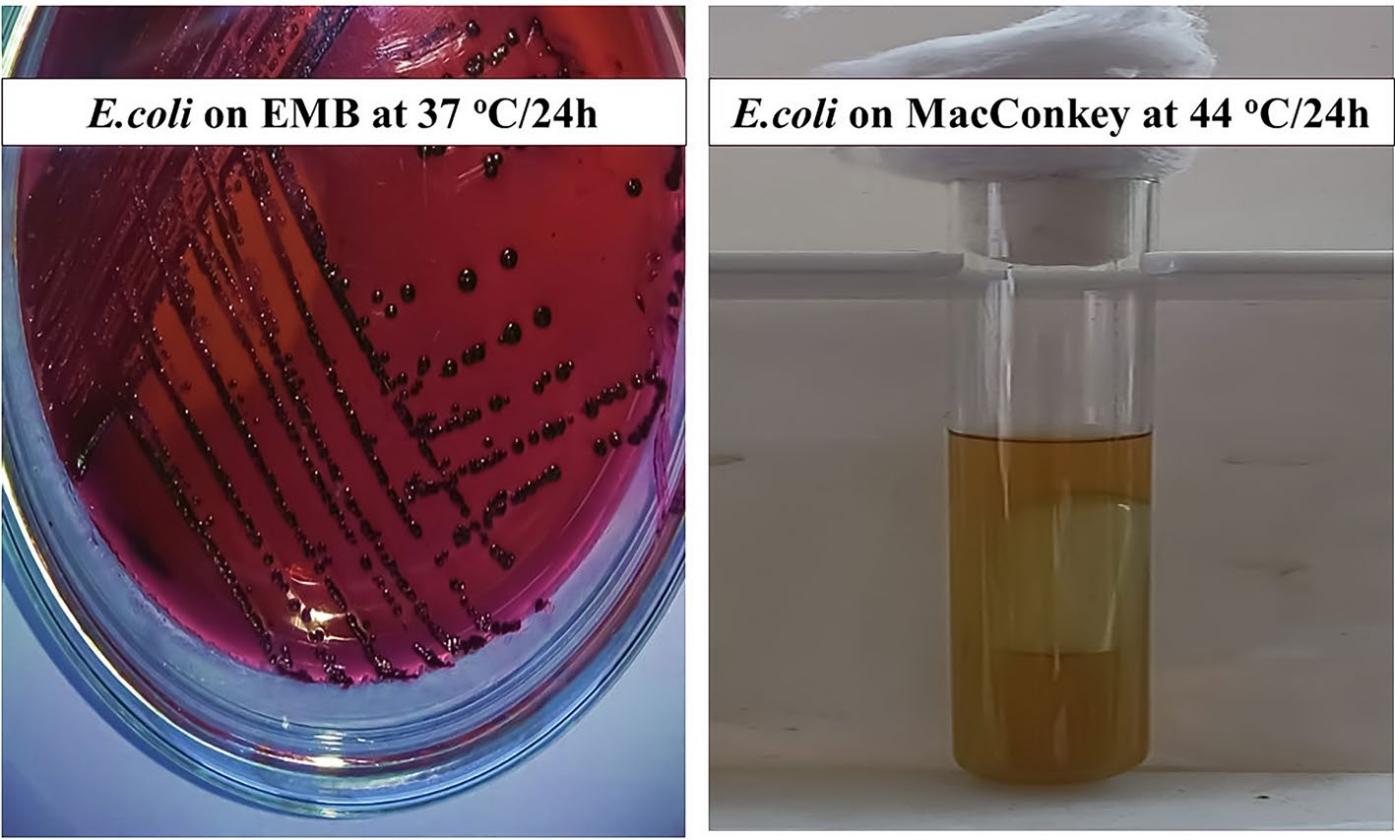
*Escherichia coli* growth on both EMB medium and MacConkey liquid medium for 24 hours at 37 °C and 44 °C, respectively.

Data in Fig. 7 proved that all *E. coli* obtained were fecal in which they had black colonies with metallic shine on EMB at 37 °C for 24 h, and had the ability to change the color of bromo cresol purple and gas production when grown on MacConkey liquid medium at 44 °C for 24 h.

#### Bioinformatics data

##### Isolation of DNA and 16s rRNA gene amplification of the most dominant pathogens affecting milk safety

For DNA analysis of the most predominant bacteria found in the feeding sterilized milk, three isolates were selected on the basis of a hemolysis test. *Staphylococcus* sp., two *Bacillus* sp. Isolates were selected for extracting of DNA to be identified based on 16srRNA analysis method.

##### Sequence analysis and distance tree construction of the most dominant pathogens affecting milk safety

**Figure 8:**
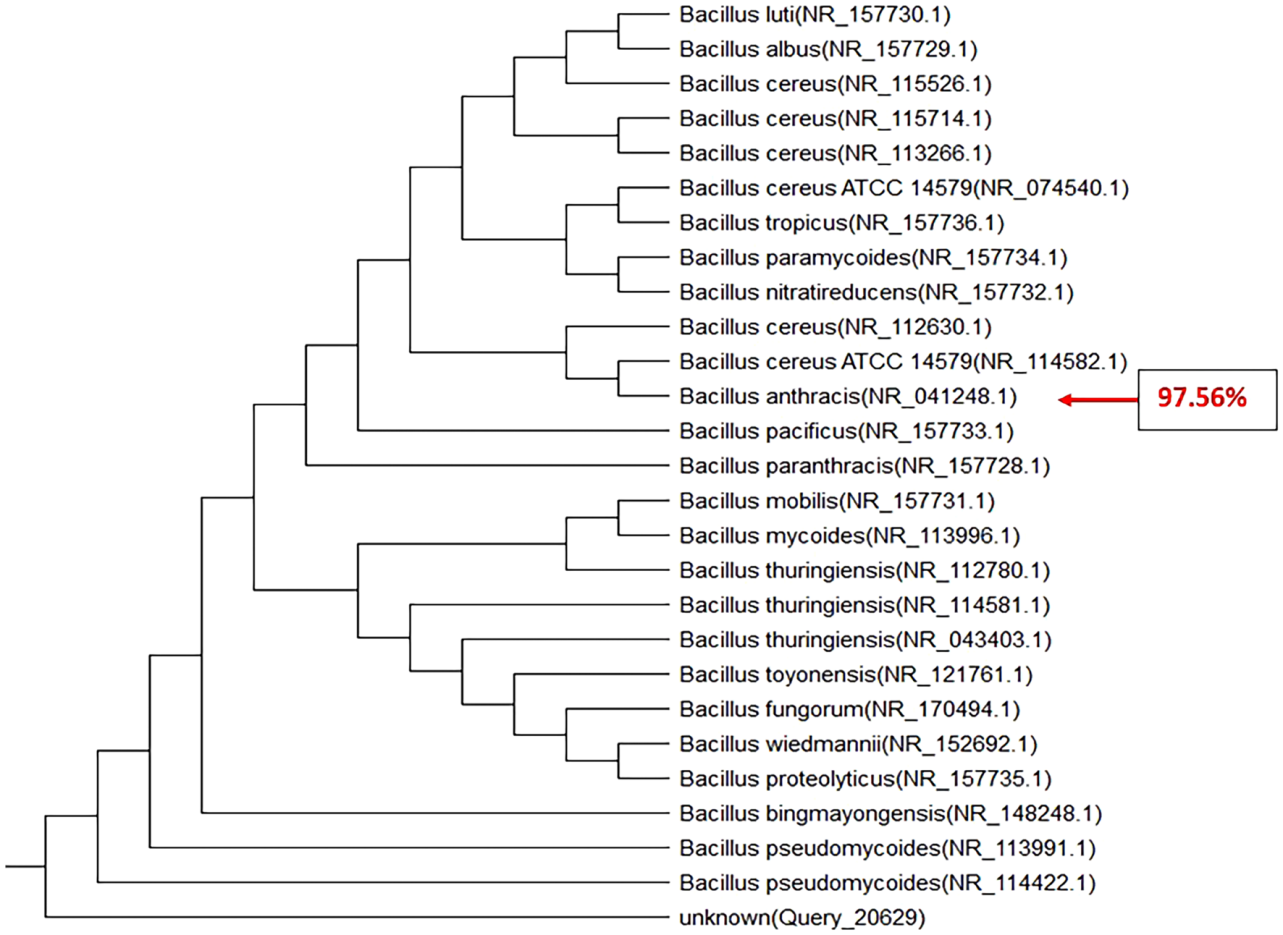
Phylogenetic tree of 16S rRNA gene sequences of *Bacillus anthracis* strain CPMESA 2021 as compared to 25 strains recorded in GenBank with gene accession number (OK585071).

**Figure 9:**
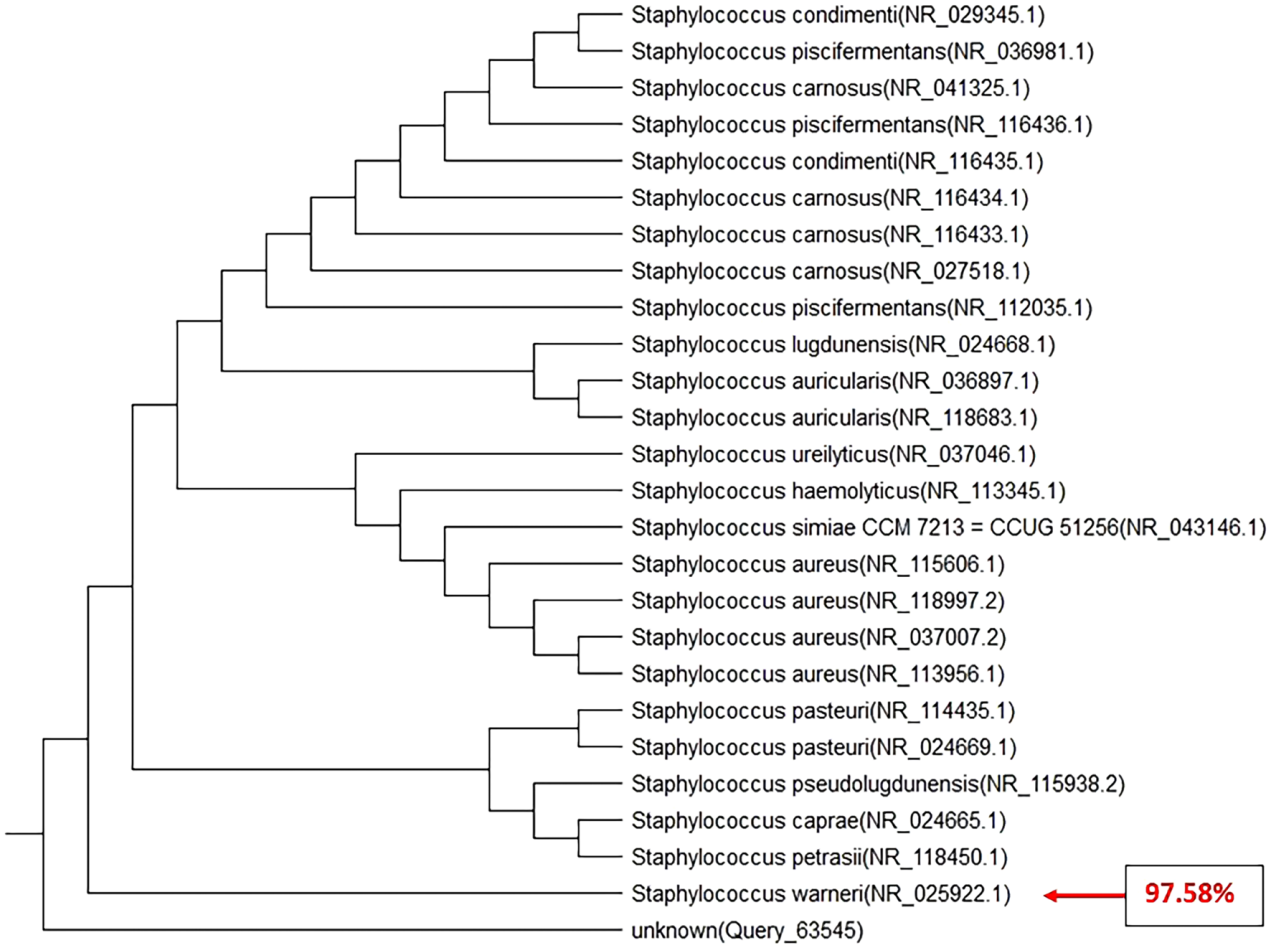
Phylogenetic tree of 16S rRNA gene sequences of *Staphylococcus warneri* strain CPSAME 2021 as compared to 25 strains recorded in GenBank with gene accession number (OK576651).

**Figure 10:**
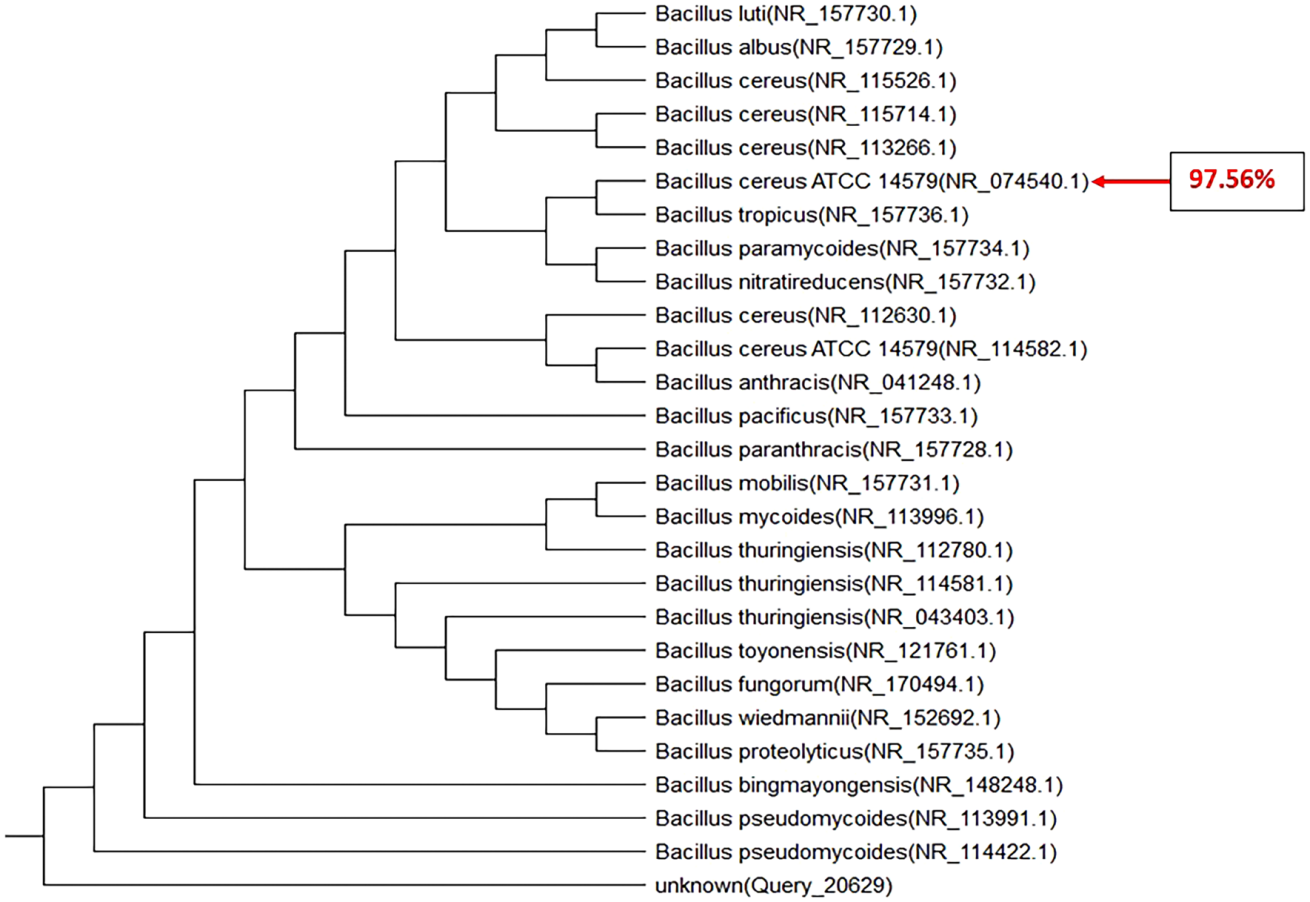
Phylogenetic tree of 16S rRNA gene sequences of *Bacillus cereus* strain CPSEMA 2021 isolate as compared to 25 strains recorded in GenBank with gene accession number (OK585052).

As mentioned above, for molecular characterization and confirmation, universal primers of 27F (5′ AGAGTTTGATCCTGGCTCAG 3′) and 1492R (5′ TACG GCTACCTTGTTACGACTT 3′) were used to amplify DNA that targets the 16S rRNA gene sequences. After DNA extraction of the isolated strains, PCR was performed with the sets of primers separately. Following the agarose gel electrophoresis, the 1500 bp and 180 bp specific amplicons of *Bacillus* and *Staphylococcus* species 16S rRNA gene were observed (Figs. 8, 9 and 10). PCR analysis confirmed the presence of the suggested isolates. These amplicons were recovered from agarose gel, TA-cloned, and sequenced. Analysis of these sequences with nucleotide BLAST revealed that they were related to the 16S rRNA gene of *Bacillus anthracis* strain CPMESA 2021, *Staphylococcus warneri* strain CPSAME 2021, and *Bacillus cereus* strain CPSEMA 2021. Their sequences were submitted to the GenBank database with the accession numbers (OK585071, OK576651, and OK585052, respectively). In the next step, these three sequences were aligned using the NCBI TREEVIEWER.

## Discussion

Food safety is a serious public health concern. Consumption of food contaminated with pathogens and microbial by-products such as toxins leads to serious diseases^31,32^. *Bacillus cereus* group represents the major food contamination sources that may lead to food poisoning which happens in the form of two types of syndromes, the emetic, and/or the diarrheal syndromes^33^. They can produce several enterotoxins: Hemolysin BL (HBL), Non-haemolytic enterotoxin (NHE) and Cytotoxin K (CytK)^34^ *B. cereus* group named also, *B. cereus *sensu lato includes eight closely related species: *B. anthracis, B. cereus *sensu stricto*, B. cytotoxicus, B. mycoides, B. pseudomycoides, B. thuringiensis, B. toyonensis*, and *B. weihenstephanensis*^35^. However, the most members recognized as pathogenic bacteria among *B. cereus* group were *B*. *cereus *sensu stricto, an opportunistic pathogen associated with food poisoning and causes soft tissue infections in humans. *Bacillus anthracis* is the causative agent of anthrax in ungulates and humans*.* It is a thermotolerant pathogen occasionally associated with food poisoning^36^. Furthermore, the level of sanitary risk appeared to be dependent on the assignment of the strain present. In addition to its pathogenicity, *B. cereus *sensu lato is an important food spoilage source because of its ability to produce many hydrolytic enzymes even at refrigerated temperatures below 7 °C which may be of concern in pasteurized foods stored at chilled temperatures^37^.

Mosquitoes are considered one of the top-ranked vectors for borne diseases. One of the most worldwide distributed mosquitoes, is *C. pipiens*^5^. In Egypt, *C. pipiens* is the historical main vector of lymphatic filariasis along the Nile Delta^10^. Studies on insect-borne bacterial and fungal diseases were neglected for many years^20^. A total of 65 bacterial and fungal isolates were isolated from the midguts of *C. pipiens* at all its developmental stages and their breeding media in nature. In this study, we tracked the incidence of eleven microbial groups inside the midguts of *C. pipiens* at different developmental stages and under different feeding strategies. Then, we tested the capability of *C. pipiens* adult^34^ females to transmit the pathogenic bacteria from their guts into the sterilized milk in the livestock farms and houses. Detection of the eleven microbial groups (*E. coli*, *Salmonella* sp., *Shigella* sp., Coliform group, *Bacillus cereus*, *Staphylococcus* sp., total bacterial count, total hemolytic count, total anaerobic bacteria, total fungi, and total yeasts) was done by growing them on specific media at the suitable incubation time and temperature. This was in line with the work done by Galal et al.^11,38^, where a total of 11 bacterial isolates have been isolated from the internal body of the various developmental stages of *C. pipiens*. But none of them has been genetically identified^38^.

Although all the eleven bacterial isolates were detected in the 4th larval instar and newly emerged female mosquitoes, only two of them were detected in newly emerged males. These results prove that the development through lifecycle of mosquitoes have an important role in the vectorial competence towards the symbiotic microorganisms. The reasons for this remain unknown but a lot of research found the same privileges of females’ mosquitoes to acquire the symbiotic microorganism from early stages like larvae^12,16–18,38^. We picked up all the isolates and maintained them for studying hemolysin production as a virulent factor. Our findings were recorded as *C. pipiens* midguts are full of various microflora at their different instars. Also, their feeding media greatly affect their midguts microbiota as the coliform group was dominant in the newly emerged adult females and larval instars, while the hemolytic bacteria were dominant in the midguts of blood and milk-fed adults. When *C. pipiens* adult females were allowed to feed on the sterilized milk, they transmitted all recorded pathogens as *E. coli*, *Coliform*, *Salmonella* sp., *B. cereus*, *Staphylococcus* sp., fungal isolates like *A. niger*, *A. flavus*, *R. nigricans*, *S. cerevisiae*, and *Rhodotorula* sp. Moreover, the predominant hemolytic bacteria were *Staphylococcus* sp. and *Bacillus* sp. We go through identifying the predominant hemolytic isolates using the 16srRNA gene analysis method and they are identified as the pathogenic *Bacillus anthracis* strain CPMESA 2021, *Staphylococcus warneri* strain CPSAME 2021, and *Bacillus cereus* strain CPSEMA 2021. Their sequences were submitted to the GenBank database with the accession numbers (OK585071, OK576651, and OK585052, respectively).

The presence of these pathogens in milk highly threatens the milk safety as an important source of dairy products for adults and children as well as puts the human’s lives at risk when they drink the infected milk without the heating processes in rural villages in Egypt. Indeed, there are heat-resistant pathogens within the detected bacteria that may cause serious illness: diarrheal and emetic diseases for people even after heating the milk^39^. All *E. coli* isolates were subjected to fecal coliform test and our data found that the obtained isolates were able to ferment lactose and form gas when incubated at 44 °C for 24 h. This proved that all obtained *E. coli* isolates were fecal isolates which transmitted from the pond water through the larvae and passed to adults through the larvae’s developmental stages and retransmitted it to the sterilized milk while feeding through their midguts^40^. According to ISO requirements in dairy product factories receiving milk shipments from live stocks, infected milk with fecal *E. coli* will subject these shipments to rejection and this loss economically affects milk producers in urban villages^41^.

Our findings correlated with earlier reports that the candidates of *Staphylococcus* sp. and *Bacillus* sp., were identified in the different organs of the mosquitoes. *Bacillus* sp. identified from the somatic tissues of mosquito *Ae. Albopictus* are capable of transmissible pathogen replication^42^. Malaria transmitting mosquitoes are frequently linked to microbes, mainly in the midgut. This may be modulating the mosquito’s vectorial capacity either by inhibition or expression through unknown mechanism. A limited number of research recorded environmental bacteria carry over in mosquitoes, but none of the research focused to know the carry over the mechanism in mosquitoes and their role in bacterial infection to the human beings^16,17,38,43,44^.

We still do not fully understand the vectorial capacity of mosquitoes for bacterial microorganisms, as well as the mechanism of transmission to the hosts and environmental feeding elements. Also, the threats of the mosquitoes as mechanical or biological vectors of diseases toward foods of human beings, are largely unknown. Mosquitoes-borne bacterial diseases are increasingly gaining the attention of medical entomologists. Although it is difficult to track the pathway of pathogens inside the internal systems of mosquitoes, scientists are trying to find answers. It is important to follow these studies in a large-scale sample to provide more evidence. The current results indicate that *C. pipiens* (a mosquito that well endemic in Egypt) has the vectorial capacity to transmit the bacterial pathogens (*Bacillus anthracis* strain CPMESA 2021, *Staphylococcus warneri* strain CPSAME 2021, and *Bacillus cereus* strain CPSEMA 2021) from their digestive system to the milk in the livestock farms and houses. These important arguments raise the question of whether *C. pipiens* could be transmitting bacterial pathogens to various human food.

## Data Availability

Accession Codes: Raw sequence data of the three selected bacterial strains were deposited in the National Center for Biotechnology Information (NCBI) under the Nucleotide section as accession numbers: OK585071 (https://www.ncbi.nlm.nih.gov/nuccore/OK585071), OK576651 (https://www.ncbi.nlm.nih.gov/nuccore/OK576651.1/) and OK585052 (https://www.ncbi.nlm.nih.gov/nuccore/OK585052), *Bacillus anthracis* strain CPMESA 2021, *Staphylococcus warneri* strain CPSAME 2021 and *Bacillus cereus* strain CPSEMA 2021, respectively.
